# Stepwise mechanism and H_2_O-assisted hydrolysis in atomic layer deposition of SiO_2_ without a catalyst

**DOI:** 10.1186/s11671-014-0714-1

**Published:** 2015-02-18

**Authors:** Guo-Yong Fang, Li-Na Xu, Lai-Guo Wang, Yan-Qiang Cao, Di Wu, Ai-Dong Li

**Affiliations:** National Laboratory of Solid State Microstructures, College of Engineering and Applied Sciences, Collaborative Innovation Center of Advanced Microstructures, Nanjing University, Nanjing, 210093 China; Zhejiang Provincial Key Laboratory of Carbon Materials, College of Chemistry and Materials Engineering, Wenzhou University, Wenzhou, 325035 China

**Keywords:** Silicon dioxide, Atomic layer deposition, H_2_O-assisted hydrolysis

## Abstract

Atomic layer deposition (ALD) is a powerful deposition technique for constructing uniform, conformal, and ultrathin films in microelectronics, photovoltaics, catalysis, energy storage, and conversion. The possible pathways for silicon dioxide (SiO_2_) ALD using silicon tetrachloride (SiCl_4_) and water (H_2_O) without a catalyst have been investigated by means of density functional theory calculations. The results show that the SiCl_4_ half-reaction is a rate-determining step of SiO_2_ ALD. It may proceed through a stepwise pathway, first forming a Si-O bond and then breaking Si-Cl/O-H bonds and forming a H-Cl bond. The H_2_O half-reaction may undergo hydrolysis and condensation processes, which are similar to conventional SiO_2_ chemical vapor deposition (CVD). In the H_2_O half-reaction, there are massive H_2_O molecules adsorbed on the surface, which can result in H_2_O-assisted hydrolysis of the Cl-terminated surface and accelerate the H_2_O half-reaction. These findings may be used to improve methods for the preparation of SiO_2_ ALD and H_2_O-based ALD of other oxides, such as Al_2_O_3_, TiO_2_, ZrO_2_, and HfO_2_.

## Background

Atomic layer deposition (ALD) is a powerful deposition technique for constructing uniform, conformal, and ultrathin films in microelectronics, photovoltaics, catalysis, energy storage, and conversion [1,2]. Compared to other fabrication techniques, such as physical vapor deposition (PVD) and chemical vapor deposition (CVD), ALD is capable of accurately controlling the thickness of thin films at the atomic scale [1]. Essentially, the principle of ALD is similar to that of CVD, except that ALD breaks the CVD reaction into two half-reactions and retains two precursors separately during the reaction [2]. Taking silicon dioxide (SiO_2_) as an example, SiO_2_ CVD using silicon tetrachloride (SiCl_4_) and water (H_2_O) can be divided into two half-reactions, A and B, of SiO_2_ ALD [3-6]:

$$ \begin{array}{c}\hfill \mathrm{C}\mathrm{V}\mathrm{D}:\mathrm{SiC}{\mathrm{l}}_4 + 2{\mathrm{H}}_2\mathrm{O}\ \to \mathrm{S}\mathrm{i}{\mathrm{O}}_2 + 4\mathrm{H}\mathrm{C}\mathrm{l},\hfill \\ {}\hfill \mathrm{A}\mathrm{L}\mathrm{D}:\left(\mathrm{A}\right)\ \mathrm{S}\mathrm{i}\hbox{-} \mathrm{O}{\mathrm{H}}^{*} + \mathrm{S}\mathrm{i}\mathrm{C}{\mathrm{l}}_4\to \mathrm{S}\mathrm{i}\hbox{-} \mathrm{O}\hbox{-} \mathrm{S}\mathrm{i}\mathrm{C}{{\mathrm{l}}_3}^{*} + \mathrm{H}\mathrm{C}\mathrm{l},\hfill \\ {}\hfill \left(\mathrm{B}\right)\ \mathrm{S}\mathrm{i}\hbox{-} \mathrm{O}\hbox{-} \mathrm{S}\mathrm{i}\mathrm{C}{\mathrm{l}}^{*} + {\mathrm{H}}_2\mathrm{O}\ \to \mathrm{S}\mathrm{i}\hbox{-} \mathrm{O}\hbox{-} \mathrm{S}\mathrm{i}\hbox{-} \mathrm{O}{\mathrm{H}}^{*} + \mathrm{H}\mathrm{C}\mathrm{l},\hfill \end{array} $$

where an asterisk designates the surface species.

**Figure 1:**
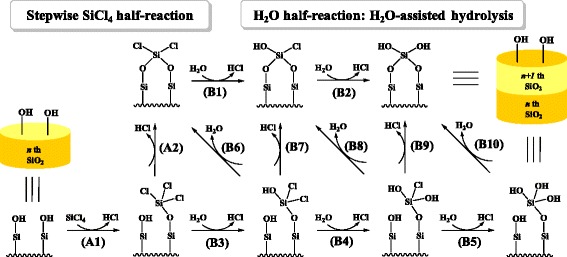
**Possible reaction mechanism of the full ALD cycle of SiO**_2_ **using SiCl**_4_ **and H**_2_ **O.** *n* represents ALD cycles.

In order to get more insight into the reaction mechanism of SiO_2_ ALD, theoretical calculation has been performed that illustrates the reaction pathways [7]. It was proposed that on the Si(001) surface, the substituted half-reaction of SiCl_4_ with surface hydroxyl group (−OH) proceeds through a concerted pathway via a four-membered ring (4MR) transition state (TS), forming Si-O and H-Cl bonds while simultaneously breaking Si-Cl and O-H bonds [7]. Unlike the Lewis acids, such as AlCl_3_, TiCl_4_, ZrCl_4_, and HfCl_4_, however, SiCl_4_ seems to have no strong nucleophilicity [8-10]. Furthermore, it was found experimentally that the rate-determining step (RDS) of the full cycle of SiO_2_ ALD is the SiCl_4_ half-reaction, not the H_2_O half-reaction [3-6]. To date, the reaction mechanism of the full SiO_2_ ALD process on the actual SiO_2_ surface has remained unclear. In this work, we have performed detailed density functional theory (DFT) calculations to investigate the reaction mechanism of the full cycle of SiO_2_ ALD, involving the SiCl_4_ half-reaction (A1 and A2) and H_2_O half-reaction (B1 to B10), as shown in Figure 1. It is demonstrated that the SiCl_4_ half-reaction may undergo a stepwise pathway and H_2_O can accelerate the H_2_O half-reaction. The insights gained into the reaction mechanism of SiO_2_ ALD may be used to improve methods for SiO_2_ ALD and H_2_O-based ALD of other oxides, such as Al_2_O_3_, TiO_2_, ZrO_2_, and HfO_2_.

## Methods

**Figure 2:**
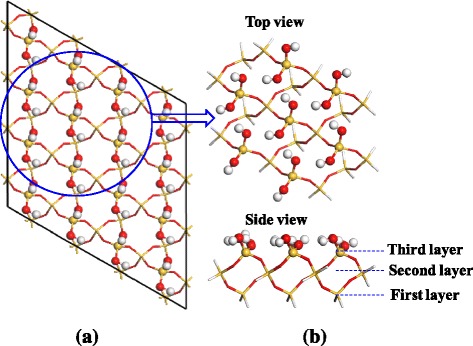
**SiO**_2_ **(0001) surface (a) and cluster model Si**_23_ **O**_40_ **H**_40_ **(b).**

In order to model the two half-reactions of SiO_2_ ALD, we adopt the cluster model Si_23_O_40_H_40_, as shown in Figure 2, which is based on a hydroxylated α-SiO_2_(0001) surface. The cluster model consists of three layers of SiO_2_ (Si_23_O_40_), and 40 hydrogen atoms which are used to saturate the dangling bonds. To stimulate the surface, the lower two layers of the SiO_2_ atoms of the two models were fixed in optimized geometries.

All the species in ALD SiO_2_ reactions were optimized using the M06-2X functional within the framework of DFT [11,12]. In order to gain a compromise between accuracy and computational cost, the 6-31G basis set was used for the fixed atoms of the substrate and the 6-31G(d,p) basis set was employed for other atoms on the surface. For each stationary point on the potential energy surface, a frequency calculation was carried out to determine if it is a minimum or a TS. All the transition states were verified by intrinsic reaction coordinates (IRC) calculations. Gibbs free energies of all species were estimated from the partition functions, and the enthalpy and entropy terms at 600 K. The energies reported here include zero-point energy (ZPE) corrections. We note that the solid surface lacks translational and rotational freedom, and the entropy of the surface only has a vibrational contribution. In other words, after being adsorbed onto the surface, the gas molecules lose translational and rotational momenta and produce new vibrational modes. All calculations in this work were performed with Gaussian 09 program [13].

## Results and discussion

### SiCl_4_ half-reaction: stepwise mechanism

**Figure 3:**
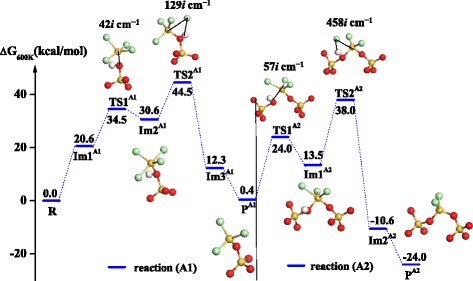
**Gibbs free energy profile of the SiCl**_4_ **half-reaction of SiO**_2_ **ALD.** The inset shows the structures of four transition states, TS1^A1^, TS2^A1^, TS1^A2^, and TS2^A2^, two pentacoordinated intermediates, Im2^A1^ and Im1^A2^, and two products, P^A1^ and P^A2^.

The reaction pathway for the SiCl_4_ half-reaction between SiCl_4_ precursor and the surface hydroxyl (-OH) is shown in Figure 3. Due to high levels of hydroxyls on the SiO_2_ surface after H_2_O half-reaction, SiCl_4_ and hydroxyl may exchange ligands twice in the SiCl_4_ half-reaction. Firstly, reaction A1 between SiCl_4_ and -OH goes through a rotation transition state, TS1^A1^, with a Gibbs free energy barrier (*G*_a_) of 34.5 kcal mol^−1^ and forms a pentacoordinated intermediate, Im2^A1^. Subsequently, the unstable intermediate undergoes the second transition state, TS2^A1^, forming the product -OSiCl_3_^*^, P^A1^ and accompanied by the release of HCl. Secondly, -OSiCl_3_ can further react with another adjacent hydroxyl (-OH) on the surface to form the bridged product -O_2_SiCl_2_^*^, P^A2^. Similar to reaction A1, reaction A2 between -OSiCl_3_^*^ and -OH also undergoes two transition states, TS1^A2^ and TS2^A2^, and a pentacoordinated intermediate, Im1^A2^. The overall SiCl_4_ half-reaction is exergonic by 24.0 kcal mol^−1^. The highest activation free energy of the SiCl_4_ half-reaction is 44.5 kcal mol^−1^ (TS2^A1^), indicating that the SiCl_4_ half-reaction is very difficult. This difficulty can be overcome by the introduction of Lewis base catalysts, such as ammonia, pyridine, and aminosilane [14-22].

In Figure 3, TS1^A1^ and TS1^A2^, with imaginary frequencies of 42*i* and 57*i* cm^−1^, respectively, represent the formation of a Si-O bond accompanied by the rotation of SiCl_4_ and -SiCl_3_. The pentacoordinated intermediates, Im2^A1^ and Im1^A2^, have a trigonal bipyramidal (TBP) geometry with five ligands of four Cl atoms and one O atom or three Cl atoms and two O atoms. The TS2^A1^ and TS2^A2^, with imaginary frequencies of 129*i* and 458*i* cm^−1^, respectively, represent cleavages of the Si-Cl and O-H bonds and the formation of a H-Cl bond. As listed in Table 1, the Si · · · O and H · · · Cl distances in reaction A1 gradually decrease from 2.01 and 2.62 Å to 1.62 and 1.29 Å, respectively, indicating the formation of new Si-O and H-Cl bonds. Simultaneously, the O-H and Si-Cl distances increase from 1.03 and 2.11 Å to 4.86 and 4.88 Å, respectively, indicating cleavage of old O-H and Si-Cl bonds. Similar to reaction A1, the Si · · · O and H · · · Cl distances in reaction A2 gradually decrease from 2.95 and 3.81 Å to 1.62 and 1.29 Å, respectively, indicating the formation of new Si-O and H-Cl bonds. Simultaneously, the O-H and Si-Cl distances increase from 0.97 and 2.05 Å to 3.44 and 4.02 Å, respectively, indicating the cleavage of these bonds.

**Table 1:** **Selected bond distances (in Å) of all species for SiCl**_4_ **half-reaction**

**Species**	**Si-O**	**O-H**	**Si-Cl**	**H-Cl**
Im1^A1^	2.01	1.03	2.11	2.62
TS1^A1^	1.83	1.08	2.24	2.83
Im2^A1^	1.79	1.13	2.24	3.21
TS2^A1^	1.77	1.02	2.94	2.01
Im3^A1^	1.62	4.86	4.88	1.29
P^A1^	2.95	0.97	2.05	3.81
TS1^A2^	2.10	1.00	2.11	2.95
Im1^A2^	1.83	1.02	2.27	2.76
TS2^A2^	1.79	1.14	2.50	1.65
Im2^A2^	1.62	3.44	4.02	1.29

### H_2_O half-reaction: H_2_O-assisted hydrolysis

In conventional SiO_2_ CVD, SiCl_4_ and H_2_O are introduced into the reaction chamber simultaneously. Subsequent hydrolysis and condensation lead to the formation of SiO_2_. Although two reactants are separately introduced into the chamber, hydrolysis and condensation also occur in SiO_2_ ALD. In fact, the half-reaction between water and the Cl-terminated surface exchanges Cl and -OH ligands and changes Si-Cl* species into Si-OH* species. Due to this the possible reactions of the H_2_O half-reaction (B) may include the formation of silanol (-Si-OH) via the exchange of ligands between Cl and -OH (reactions B1, B2, B3, B4, and B5) and the formation of -O-Si-O- bridge bonds by removing H_2_O (reactions B6, B8, and B10) and HCl (reactions B7 and B9), similar to the hydrolysis (-Si-OH) and condensation (-O-Si-O-) processes of SiO_2_ CVD.

**Figure 4:**
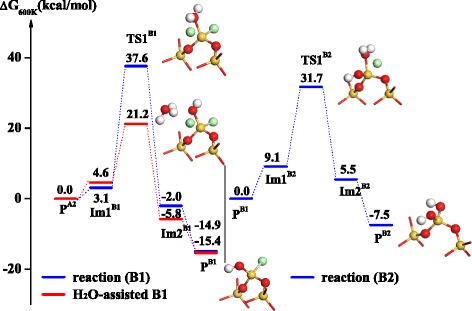
**Gibbs free energy profiles of the hydrolysis reactions of -O**_2_ **SiCl**_2_^*^ **, B1 and B2, in H**_2_ **O half-reaction.** The inset shows the structures of three transition states, TS1^B1^, TS1^B2^, and H_2_O-assisted-TS1^B1^, and two products, P^B1^ and P^B2^.

After the SiCl_4_ half-reaction, the hydroxylated surface is terminated by Cl atoms and changes to -O_2_SiCl_2_^*^ and -OSiCl_3_^*^ surfaces, which are both hydrolyzed in subsequent H_2_O half-reaction. Firstly, H_2_O and the bridged surface (-O_2_SiCl_2_^*^) can exchange the ligands via reactions B1 and B2, shown in Figure 4. The first Cl exchange of the hydrolysis of -O_2_SiCl_2_^*^ requires a high activation free energy of 37.6 kcal mol^−1^ and goes through a transition state, TS1^B1^, to form -Si-OH^*^ species and release HCl from the surface. Subsequently, the second Cl atom of -O_2_SiCl_2_^*^ can also be exchanged by -OH via a transition state, TS1^B2^, with an activation free energy of 31.7 kcal mol^−1^. If H_2_O-assisted role is considered, the activation free energy of the hydrolysis of -O_2_SiCl_2_^*^ decreases to approximately 21.2 kcal mol^−1^, indicating that H_2_O can accelerate -Si-OH formation and Cl elimination. The reason for this is mainly that H_2_O can form hydrogen bonding interactions through H_2_O…H_2_O bonds and lower the activation energy of Si-O bond formation and Cl elimination via a six-member ring (6MR) transition state, H_2_O-assisted-TS1^B1^, shown in Figure 4. The accelerated half-reaction via the hydrogen bonding interaction of H_2_O…H_2_O may be termed as H_2_O-assisted hydrolysis, which is similar to Lewis-base catalysis in SiO_2_ ALD through the OH…N hydrogen bond [14,23,24]. As a matter of fact, there are H_2_O-assisted reactions in nature, such as hydrolysis or solvolysis [25-28], tautomerization or proton transfer [29-34], decomposition [35-37], and catalysis [38,39]. H_2_O-assisted hydrolysis and solvolysis facilitate the exchange and dissociation of Cl ligand in HfO_2_ ALD using HfCl_4_ and H_2_O [40].

**Figure 5:**
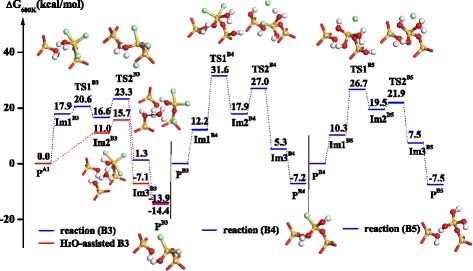
**Gibbs free energy profiles of the hydrolysis reactions of -OSiCl**_3_^*^ **, B3, B4, and B5, in H**_2_ **O half-reaction.** The inset shows the structures of seven transition states, TS1^B3^, TS2^B3^, TS1^B4^, TS2^B4^, TS1^B5^, TS2^B5^, and H_2_O-assisted**-**TS2^B3^, a pentacoordinated intermediate, H_2_O-assisted-Im2^B3^, and three products, P^B3^, P^B4^, and P^B5^.

Secondly, another Cl-terminated surface (-OSiCl_3_^*^) can also hydrolyze step-by-step and go through pathways, B3, B4, and B5, shown in Figure 5. Three ligand exchange reactions undergo two transition states, TS1^B3^ and TS2^B3^, TS1^B4^ and TS2^B4^, and TS1^B5^ and TS2^B5^. Similar to the SiCl_4_ half-reaction, the first represents the formation of Si-O bonds and the second represents the cleavages of Si-Cl and O-H bonds and the formation of H-Cl bond. It is found that the activation free energies of -OSiCl_3_^*^ hydrolysis are lower than that of -O_2_SiCl_2_^*^ hydrolysis. Unlike the rigid -O_2_SiCl_2_ group, the -OSiCl_3_ group is more flexible. As shown in TS1^B3^ of Figure 5, the hydroxyl (-OH) on the surface can interact with H_2_O through hydrogen bonding, HOH…OH, and cause the rotation of the -OSiCl_3_ group, which can accelerate the hydrolysis of -OSiCl_3_ and H_2_O exchange with the Cl ligand. The first hydrolysis of -OSiCl_3_^*^ requires a low activation free energy of 23.3 kcal mol^−1^; however, the hydrolysis of -OSiOH-Cl_2_^*^ and -OSi(OH)_2_-Cl^*^ require slightly higher activation free energies. The reason for this may be that the direction of the hydrolyzed Cl atom of -OSiCl_3_^*^ is more downward than that of -OSiOH-Cl_2_^*^ or -OSi(OH)_2_-Cl^*^, which results in a hydrogen bonding interaction between -OH and H_2_O. In the H_2_O half-reaction, there are massive H_2_O molecules adsorbed on the surface, which result in H_2_O-assisted hydrolysis. Owing to the strong hydrogen bonding interaction of H_2_O · · · H_2_O, a pentacoordinated intermediate including silanol (Si-OH) ligand can be directly formed, as shown in H_2_O-assisted Im2^B3^ in Figure 5. The hydrolysis of -OSiCl_3_^*^ and the elimination of Cl ligand occur easily and the activation free energy can decrease from 23.3 to 15.0 kcal mol^−1^. Similarly, H_2_O can also accelerate the hydrolysis of -OSiOH-Cl_2_^*^ and -OSi(OH)_2_-Cl^*^.

**Figure 6:**
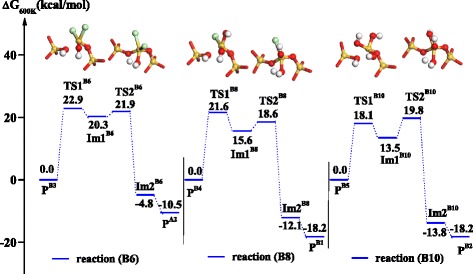
**Gibbs free energy profiles of H**_2_ **O condensation reactions, B6, B8, and B10, in H**_2_ **O half-reaction.** The inset shows the structures of six transition states, TS1^B6^, TS2^B6^, TS1^B8^, TS2^B8^, TS1^B10^, and TS2^B10^.

As show in Figure 6, the formation of the O-Si-O bridge bond can result from H_**2**_O condensation reactions, B6, B8, and B10, similar to the condensation (O-Si-O) process of SiO_2_ CVD. These condensation reactions occur after the hydrolysis of -OSiCl_3_^*^, -OSiOH-Cl_2_^*^ and -OSi(OH)_2_-Cl^*^ and include two transition states. The first transition states, TS1^B6^, TS1^B8^, and TS1^B10^, represent O-Si-O bond formation with the activation free energies of 22.9, 21.6, and 18.1 kcal mol^−1^, respectively. The second transition states, TS2^B6^, TS2^B8^, and TS2^B10^, represent H_2_O removal with the activation free energies of 21.9, 18.6, and 19.8 kcal mol^−1^, respectively.

**Figure 7:**
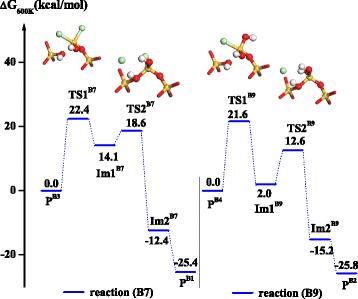
**The Gibbs free energy profiles of HCl condensation reactions, B7 and B9, in H**_2_ **O half-reaction.** The inset shows the structures of four transition states, TS1^B7^, TS2^B7^, TS1^B9^, and TS2^B9^.

Similar to H_2_O condensation, HCl condensation reactions, B7 and B9, can also result in the formation of the O-Si-O bridge bond with low activation free energies of 22.4 and 21.6 kcal mol^−1^, respectively, as show in Figure 7. The corresponding activation free energies of HCl removal are 18.6 and 12.6 kcal mol^−1^, respectively. During H_2_O or HCl removal, the two condensations both lead to the formation of the O-Si-O bridge bond, which is the elementary unit of SiO_2_ and ensures its ALD growth.

When reviewing the full SiO_2_ ALD cycle, including reactions A1 to A2 and B1 to B10, we find that the free energy barrier for the H_2_O half-reaction is lower than that for SiCl_4_ half-reaction. The principal reason is that there are massive H_2_O molecules adsorbed on the surface, which result in H_2_O-assisted hydrolysis of -O_2_Si-Cl_2_^*^, -O_2_SiOH-Cl^*^, -OSi-Cl_3_^*^, -OSiOH-Cl_2_^*^, and -OSi(OH)_2_-Cl^*^ and accelerate the H_2_O half-reaction. Therefore, the SiCl_4_ half-reaction is the RDS of the full ALD cycle of SiO_2_ and controls the ALD growth of SiO_2_.

## Conclusions

Through detailed DFT calculations, the possible reaction pathways of (A) SiCl_4_ half-reaction and (B) H_2_O half-reaction in SiO_2_ ALD without a catalyst have been investigated. The SiCl_4_ half-reaction is the RDS of SiO_2_ ALD. It may proceed through a stepwise pathway, first forming a Si-O bond and then breaking Si-Cl and O-H bonds and forming a H-Cl bond. The H_2_O half-reaction is a complicated process, including hydrolysis and condensation. In the H_2_O half-reaction, there are massive H_2_O molecules adsorbed on the surface, which can result in H_2_O-assisted hydrolysis of the Cl-terminated surface and accelerate the H_2_O half-reaction. These findings may be used in SiO_2_ ALD and H_2_O-based ALD of other oxides, such as Al_2_O_3_, TiO_2_, ZrO_2_, and HfO_2_.

## References

[1] Doering R, Nishi Y (2007). Handbook of semiconductor manufacturing technology.

[2] Pinna N, Knez M (2011). Atomic layer deposition of nanostructured materials.

[3] George SM, Sneh O, Dillon AC, Wise ML, Ott AW, Okada LA (1994). Atomic layer controlled deposition of SiO_2_ and Al_2_O_3_ using ABAB… binary reaction sequence chemistry. Appl Surf Sci.

[4] Sneh O, Wise ML, Ott AW, Okada LA, George SM (1995). Atomic layer growth of SiO_2_ on Si(100) using SiCl_4_ and H_2_O in a binary reaction sequence. Surf Sci.

[5] George SM, Ott AW, Klaus JW (1996). Surface chemistry for atomic layer growth. J Phys Chem.

[6] Klaus JW, Ott AW, Johnson JM, George SM (1997). Atomic layer controlled growth of SiO_2_ films using binary reaction sequence chemistry. Appl Phys Lett.

[7] Kang JK, Musgrave CB (2002). Mechanism of atomic layer deposition of SiO_2_ on the silicon (100)-2 × 1 surface using SiCl_4_ and H_2_O as precursors. J Appl Phys.

[8] Ritala M, Kukli K, Rahtu A, Räisänen PI, Leskelä M, Sajavaara T (2000). Atomic layer deposition of oxide thin films with metal alkoxides as oxygen sources. Science.

[9] Hausmann D, Becker J, Wang S, Gordon RG (2002). Rapid vapor deposition of highly conformal silica nanolaminates. Science.

[10] Fang G, Ma J (2013). Rapid atomic layer deposition of silica nanolaminates: synergistic catalysis of Lewis/Brønsted acid sites and interfacial interactions. Nanoscale.

[11] Zhao Y, Truhlar DG (2008). Density functionals with broad applicability in chemistry. Acc Chem Res.

[12] Zhao Y, Truhlar DG (2008). The M06 suite of density functionals for main group thermochemistry, thermochemical kinetics, noncovalent interactions, excited states, and transition elements: two new functionals and systematic testing of four M06-class functionals and 12 other functional. Theor Chem Acc.

[13] Frisch MJ, Trucks GW, Schlegel HB, Scuseria GE, Robb MA, Cheeseman JR (2009). Gaussian 09. Revision B.02.

[14] Klaus JW, Sneh O, George SM (1997). Growth of SiO_2_ at room temperature with the use of catalyzed sequential half reaction. Science.

[15] Klaus JW, Sneh O, Ott AW, George SM (1999). Atomic layer deposition of SiO_2_ using catalyzed and uncatalyzed self-limiting surface reactions. Surf Rev Lett.

[16] Klaus JW, George SM (2000). Atomic layer deposition of SiO_2_ at room temperature using NH_3_-catalyzed sequential surface reactions. Surf Sci.

[17] Klaus JW, George SM (2000). SiO_2_ chemical vapor deposition at room temperature using SiCl_4_ and H_2_O with an NH_3_ catalyst. J Electrochem Soc.

[18] Ferguson JD, Smith ER, Weimer AW, George SM (2004). ALD of SiO_2_ at room temperature using TEOS and H_2_O with NH_3_ as the catalyst. J Electrochem Soc.

[19] Du Y, Du X, George SM (2005). SiO_2_ film growth at low temperatures by catalyzed atomic layer deposition in a viscous flow reactor. Thin Sol Film.

[20] Du Y, Du X, George SM (2007). Mechanism of pyridine-catalyzed SiO_2_ atomic layer deposition studied by Fourier transform infrared spectroscopy. J Phys Chem C.

[21] Hatton B, Kitaev V, Perovic D, Ozin G, Aizenberg J (2010). Low-temperature synthesis of nanoscale silica multilayers - atomic layer deposition in a test tube. J Mater Chem.

[22] Bachmann J, Zierold R, Chong YT, Hauert R, Sturm C, Schmidt-Grund R (2008). A practical, self-catalytic, atomic layer deposition of silicon dioxide. Angew Chem Int Ed.

[23] Fang G, Chen S, Li A, Ma J (2012). Surface pseudorotation in Lewis-base-catalyzed atomic layer deposition of SiO_2_: static transition state search and Born − Oppenheimer molecular dynamics simulation. J Phys Chem C.

[24] Fang GY, Xu LN, Cao YQ, Wang LG, Wu D, Li DL (2015). Self-catalysis by aminosilanes and strong surface oxidation by O_2_ plasma in plasma-enhanced atomic layer deposition of high-quality SiO_2_. Chem Commun.

[25] Antonczak S, Ruiz-Lόpez MF, Rivail JL (1994). Ab initio analysis of water-assisted reaction mechanisms in amide hydrolysis. J Am Chem Soc.

[26] Schmeer G, Sturm P (1999). A quantum chemical approach to the water assisted neutral hydrolysis of ethyl acetate and its derivatives. Phys Chem Chem Phys.

[27] Tsuchida N, Satou H, Yamabe S (2007). Reaction paths of the water-assisted solvolysis of N, N-dimethylformamide. J Phys Chem A.

[28] Gao JY, Zeng Y, Zhang CH, Xue Y (2009). Theoretical studies on the water-assisted hydrolysis of N, N-dimethyl-N’-(2′,3′-dideoxy-3′-thiacytidine) formamidine with three water molecules. J Phys Chem A.

[29] Bell RL, Truong TN (1997). Primary and solvent kinetic isotope effects in the water-assisted tautomerization of formamidine: an ab initio direct dynamics study. J Phys Chem A.

[30] Gu J, Leszczynski J (1999). A DFT study of the water-assisted intramolecular proton transfer in the tautomers of adenine. J Phys Chem A.

[31] Liu GX, Li ZS, Ding YH, Fu Q, Huang XR, Sun CC (2002). Water-assisted isomerization from linear propargylium (H_2_CCCH^+^) to cyclopropenylium(c-C_3_H_3_^+^). J Phys Chem A.

[32] Balta B, Aviyente V (2004). Solvent effects on glycine II. Water-assisted tautomerization. J Comput Chem.

[33] Markova N, Enchev V, Timtcheva I (2005). Oxo-hydroxy tautomerism of 5-fluorouracil: water-assisted proton transfer. J Phys Chem A.

[34] Michalkova A, Kosenkov D, Gorb L, Leszczynski J (2008). Thermodynamics and kinetics of intramolecular water assisted proton transfer in Na^+^-1-methylcytosine water complexes. J Phys Chem B.

[35] Aplincourt P, Anglada JM (2003). Theoretical studies of the isoprene ozonolysis under tropospheric conditions. 2. Unimolecular and water-assisted decomposition of the r-hydroxy hydroperoxides. J Phys Chem A.

[36] Jacobs G, Patterson PM, Graham UM, Crawford AC, Dozier A, Davis BH (2005). Catalytic links among the water–gas shift, water-assisted formic acid decomposition, and methanol steam reforming reactions over Pt-promoted thoria. J Cat.

[37] Huang J, Yeung CS, Ma J, Gayner ER, Phillips DL (2014). A computational chemistry investigation of the mechanism of the water-assisted decomposition of trichloroethylene oxide. J Phys Chem A.

[38] Ouchi M, Yoda H, Terashima T, Sawamoto M (2012). Aqueous metal-catalyzed living radical polymerization: highly active water-assisted catalysis. Polym J.

[39] Thorat PB, Goswami SV, Jadhav WN, Bhusare SR (2013). Water-assisted organocatalysis: an enantioselective green protocol for the henry reaction. Aust J Chem.

[40] Mukhopadhyay AB, Musgrave CB, Sanz JF (2008). Atomic layer deposition of hafnium oxide from hafnium chloride and water. J Am Chem Soc.

